# Synchronous perforation of non-Hodgkin's lymphoma of the small intestine and colon: a case report

**DOI:** 10.1186/1752-1947-5-57

**Published:** 2011-02-10

**Authors:** Mohamad S Dughayli, Fadi Baidoun, Aaron Lupovitch

**Affiliations:** 1 Department of Surgery, Henry Ford Wyandotte Hospital Wyandotte, Michigan, USA; 2 Department of Pathology, Henry Ford Wyandotte Hospital, Wyandotte, Michigan, USA

## Abstract

**Introduction:**

Primary non-Hodgkin's lymphoma of the small and large bowel presenting as a perforated viscus entity with peritonitis is extremely rare. A thorough literature review did not reveal any cases where primary lymphoma of the jejunum presented with perforation and peritonitis synchronously with primary lymphoma of the descending colon.

**Case presentation:**

This report concerns a 64-year-old Caucasian woman admitted with severe abdominal pain and fever. An emergency laparotomy revealed a large mass with perforation in the proximal jejunum with intense mesenteric thickening and lymphadenopathy. The descending colon was edematous and covered with fibrinous exudate. Histopathological examination of the resected segment of jejunum revealed a T cell non-Hodgkin's lymphoma. On post-operative day 10, a computed tomography scan of our patient's abdomen and pelvis showed leakage of contrast into the pelvis. Re-exploration revealed perforation of the descending colon. The histopathology of the resected colon also showed T cell non-Hodgkin's lymphoma. Her post-operative course was complicated by acute renal and respiratory failure. The patient died on post-operative day 21.

**Conclusions:**

Lymphoma of the small intestine has been reported to have a poor prognosis. The synchronous occurrence of lesions in the small intestine or colon is unusual, and impacts the prognosis adversely. Early diagnosis and treatment are important to improve the prognosis of bowel perforation in patients with non-Hodgkin's lymphoma.

## Introduction

Despite the fact that the small bowel represents 75% of the length and over 90% of the mucosal surface of the intestinal tract, malignant tumors of the small bowel account for less than 1% of intestinal malignances and primary lymphomas of the small intestine are rare [1,2].

T cell lymphomas (TCL) have higher incidence rates in Asia than in Western countries [3]. These tumors have been described as a specific type in a proposal for a revised European-American classification of lymphoid neoplasms [4]. Retrospective analysis [5] has indicated that in Western populations, 60% to 80% of intestinal lymphomas are B cell lymphomas. Intestinal TCLs have been described as often being multifocal and most frequently localized in the proximal ileum and jejunum [6]. TCLs involving the colon are rare and account for only 4% to 6% of gastrointestinal lymphomas [7]. Because of their rarity, non-specific symptoms and diagnostic difficulties, small bowel tumors are often diagnosed and treated late in their course. The diagnostic difficulty is increased when these tumors arise in association with primary synchronous tumors of the colon.

A thorough literature review using Medline did not reveal any previously reported cases where primary lymphoma of the jejunum had presented with perforation and peritonitis synchronously with a primary lymphoma of the descending colon.

## Case presentation

A 64-year-old Caucasian woman presented to our emergency room with severe abdominal pain of four days duration, associated with fever and chills in the last 24 hours. Our patient had an eight-month history of vague abdominal pain, anemia, weight loss, and change in bowel habits. She underwent extensive investigation for non-specific abdominal pain and no pathology was found. This investigation included blood tests, esophagogastroduodunoscopy, colonoscopy, and a computed tomography (CT) scan of the abdomen and pelvis. Her surgical history was significant for a donor left nephrectomy.

**Figure 1:**
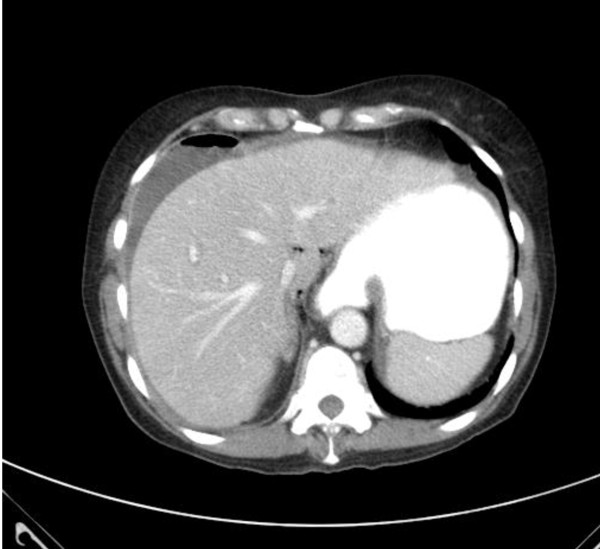
**A computed tomography (CT) scan of the abdomen and pelvis showing free intra-peritoneal air consistent with a perforated viscus**.

A physical examination conducted in the emergency room revealed she was in acute distress, with a distended abdomen and peritonitis. The laboratory test results showed a white blood cell (WBC) count of 15,700/mm^3, neutrophils 87%, lymphocytes 6%, Na 137 mEq/L, K 3.5 mEq/L, Cr 1.2 mg/dL, and albumin 2.3 g/dL. A CT scan of the abdomen and pelvis showed a large collection of contrast media in the left upper quadrant associated with multiple small pockets of air, suggestive of perforation most likely in the proximal small bowel (Figure 1).

**Figure 2:**
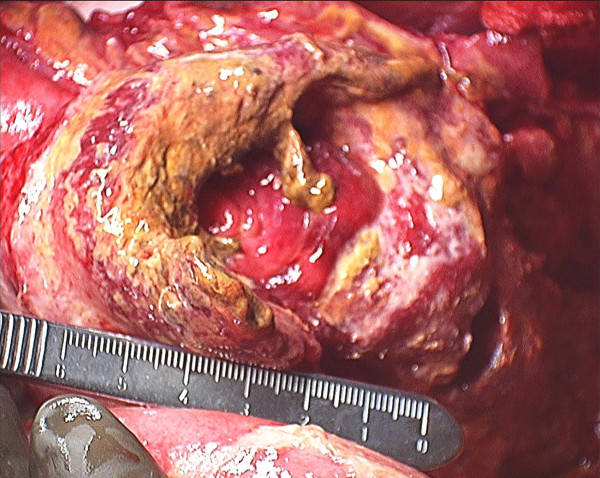
**Perforation of the proximal jejunum**.

On laparotomy, copious amount of fluid, intestinal contents and well-organized pus (collection of pus surrounded by a capsule) were found in the left upper quadrant of the peritoneal cavity. Upon exploration we found a perforated tumor in the proximal jejunum measuring 8 × 5 cm in size, positioned 10 cm from the ligament of Treitz (Figure 2). The proximal jejunum was edematous with thickened inflamed mesentery and enlarged lymph nodes. The left descending colon was edematous and covered with flakes of pus and exudates. A small bowel resection was performed with Roux-en-Y retrocolic gastrojejunostomy, gastrostomy and duodenostomy tube placement.

**Figure 3:**
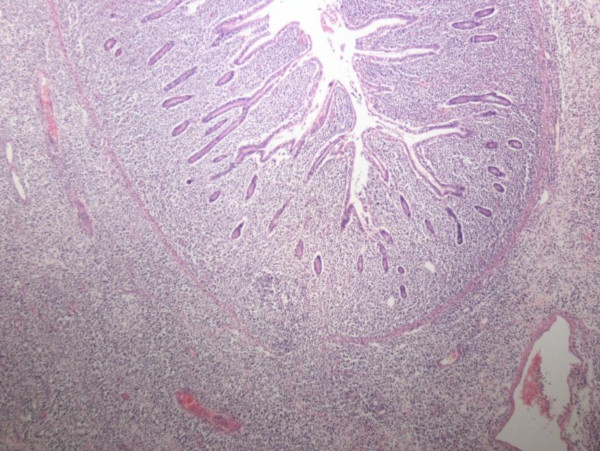
**T cell lymphoma infiltrating all layers of the jejunal wall**. Hematoxylin and eosin stain, magnification 40 ×.

**Figure 4:**
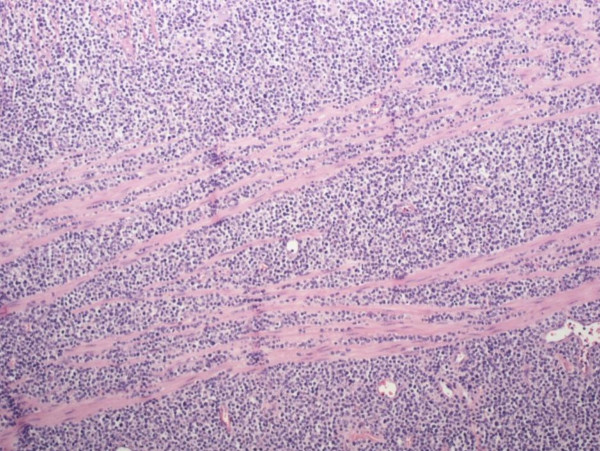
**Homogeneous infiltrate of T cells separating fibers of the muscularis propria**. Hematoxylin and eosin stain, magnification 100 ×.

**Figure 5:**
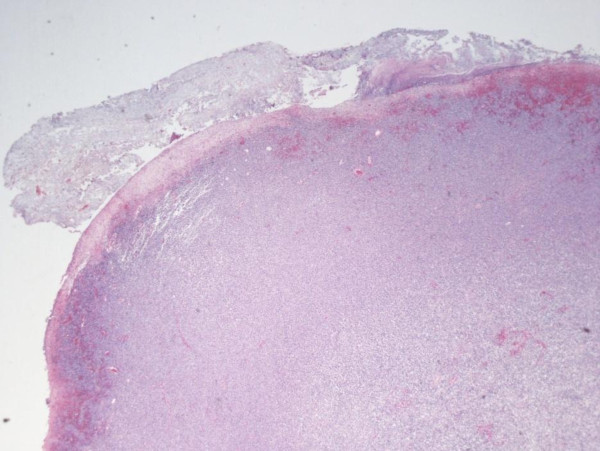
**Transmural necrotic tract through the intestinal wall and lymphoma**. Fecal content overlies the zone of necrosis and coats the tract lumen, indicating expulsion into the peritoneal cavity. Hematoxylin and eosin stain, magnification 20 ×.

Results of a mesenteric lymph node sample sent for intra-operative consultation revealed probable lymphoma. The segments of resected small intestine had a combined length of 22 cm, and a circumference of 8 cm. A 5 cm-long area of transmural bowel necrosis with perforation was present. The adjacent bowel wall was inflamed and thickened. No grossly discerned mass was noted. Light microscopic findings, immunohistochemical staining and testing for T cell gene rearrangement indicated a T cell non-Hodgkin's lymphoma extending through the full thickness of the bowel wall with an area of transmural necrosis and gross perforation (Figures 3, 4, 5).

**Figure 6:**
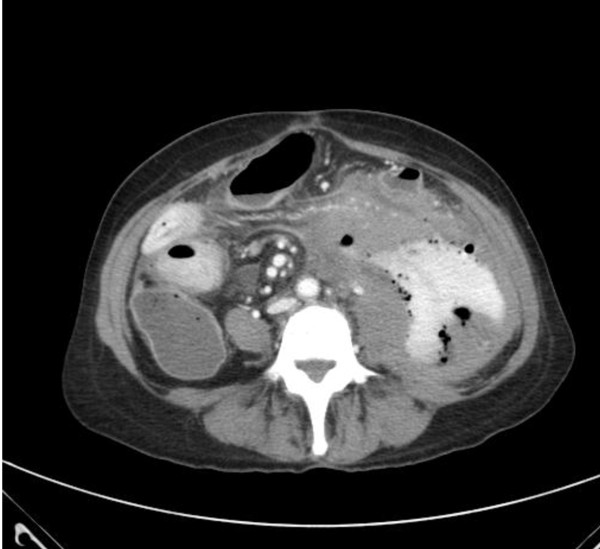
**A computed tomography (CT) scan of the abdomen and pelvis showing loculated extravasation of contrast and air consistent with a ruptured viscus**.

After the operation, our patient was transferred to our intensive care unit in a stable condition. On post-operative day one, she was extubated and started on total parenteral nutrition. Then, two days later, due to respiratory distress our patient was reintubated and started on enteral tube feeding, after which she had bowel movements and was doing relatively well. On post-operative day 9, her clinical condition began to deteriorate and she was diagnosed with sepsis. A repeat CT scan of the abdomen and pelvis revealed leakage into the abdominal cavity (Figure 6). Urgent re-exploration of the abdominal cavity revealed a moderate amount of contrast material and intestinal contents in the left paracolic gutter. A perforation in the descending colon was noted. A left colectomy with transverse colostomy was performed.

The resected segment of colon had the same clinical and pathological features of the jejunum. After the second laparatomy our patient's condition continued to deteriorate, with respiratory and renal failure. Subsequently, her family decided to pursue palliative care only, and our patient died after withdrawal of care.

## Discussion

Our report describes an unusual case of simultaneous small and large bowel non-Hodgkin's lymphoma with synchronous perforation. Tumors of the small intestine are infrequent; only 3% to 6% of gastrointestinal tumors and 1% of gastrointestinal malignances arise from the small bowel [8]. They are more common in the ileum, consistent with the higher number of lymphocytes there [9]. The frequency varies according to the geographic location and ethnic origin of the population [10]. T cell lymphomas have a lower incidence in Western countries [11]. Lymphoma is the commonest malignant disease occurring as a complication of celiac disease. Loughran *et al*. have also found T cell lymphomas in patients with a long history of celiac disease in the small bowel and ulcerative colitis in the colon [12]. The colon itself is an uncommon site of involvement in non-Hodgkin's lymphoma. Zighelboim and Larson [13] analyzed their 19-year experience at the Mayo clinic and found that the most common site of involvement was the cecum, 73%, followed by the rectum. Doolabh and colleagues [14] reported similar rates of cecal involvement and found that the lack of specific symptoms delayed diagnosis by one to 12 months.

The typical patient is in their fifth or sixth decade of life. The most common presenting symptoms include abdominal pain, altered bowel habits and weight loss that in some patients had persisted for months before a diagnosis was made. Other symptoms include bleeding, obstruction, perforation, and intussusceptions. No sex predominance exists. Some patients remain asymptomatic until intestinal perforation. At diagnosis the lymphoma was bulky in 65% of patients, reaching over 10 cm in 45% of cases, implying a delay in diagnosis with a possibly adverse effect on prognosis. The lack of specific complaints and the rarity of intestinal obstruction probably account for the delays in diagnosis. Early diagnosis and systemic chemotherapy may prevent the occurrence of perforation and the need for surgery.

Proposal of the Revised European and American Lymphoma (REAL) classification in 1994 generated new interest in T cell lymphomas. According to this classification, peripheral T cell lymphomas (PTCLs) are a subset of T cell lymphomas [3]. PTCL is diagnosed when tumor cells express the mature T cell antigens CD2, CD3, CD4, CD5, CD6, or CD7 on immunohistochemical staining [15]. The site of origin and clinical manifestation are also important factors in the definition of a specific clinicopathological entity of PTCL. Reported cases were mostly associated with enteropathy such as celiac disease. Intestinal T cell lymphoma usually involves the jejunum and has multiple ulcerations, often with perforation [3]. Our patient did not have associated enteropathy but did exhibit the histological features of angiocentric invasion; we think that the lymphoma in our patient might be best classified as primary intestinal lymphoma involving the proximal jejunum and descending colon.

Treatment generally includes surgery, radiation, therapy, and chemotherapy. In the treatment of high-grade intestinal T cell non-Hodgkin's lymphoma or anaplastic large cell type lymphoma, a multimodality approach is superior to surgery or chemotherapy alone. Prognostic factors include the stage at presentation, the presence of perforation, tumor resectability, histological subtype, and the use of multimodality therapy. Perforated lymphomas usually have higher tumor staging and poorer prognosis.

The clinical evolution of T cell lymphoma is aggressive, and the five-year survival rate is 25% [16]. The morbidity and mortality of intestinal lymphoma presenting with perforation is high, as the perforation may go unrecognized until shock follows peritonitis. The time interval from the onset of symptoms caused by the perforation to the time of operation can affect the outcome. Pre-operative shock is also a significant poor prognostic factor for such patients.

## Conclusions

Surgeons should always be alert for the possibility of multiple sites of malignancy during laparotomy. T cell non-Hodgkin's lymphoma is a rapidly progressive malignancy that may present with surgical complications. Poor prognostic factors include advanced age, late stage disease, and a poor performance status, as well as delay and contraindication of chemotherapy. The prognosis of synchronous primary lymphoma in the small and large bowel correlates better with the depth of invasion, tumor size, and lymphadenopathy. In our patient the prognosis was always poor, especially because of the complicated post-operative course with a second perforation in the descending colon. We speculate that our patient's outcome may have been different if the lesion in the descending colon was diagnosed in the first setting and if chemotherapy had been feasible in the early post-operative course.

## Consent

Written informed consent was obtained from the patient's next-of-kin for publication of this case report and any accompanying images. A copy of the written consent is available for review by the Editor-in-Chief of this journal.

## Competing interests

The authors declare that they have no competing interests.

## Authors' contributions

All authors read and approved the final manuscript. MD reviewed the literature and participated in writing the abstract, introduction, and discussion sections. FB participated in writing the case report and prepared the figures. AL wrote the pathology section and prepared the pathology figures.
